# The Predictive and Prognostic Nature of Programmed Death-Ligand 1 in Malignant Pleural Mesothelioma: A Systematic Literature Review

**DOI:** 10.1016/j.jtocrr.2022.100315

**Published:** 2022-03-22

**Authors:** Aaron S. Mansfield, Rebecca J. Brown, Cormac Sammon, Melinda J. Daumont, Mike McKenna, Jenine K. Sanzari, Patrick M. Forde

**Affiliations:** a Division of Medical Oncology, Mayo Clinic, Rochester, Minnesota; b PHMR Ltd., Berkeley Works, London, United Kingdom; c Bristol-Myers Squibb, Braine-l’Alleud, Belgium; d Health Outcomes Solutions Ltd., London, United Kingdom; e Bristol-Myers Squibb, Lawrenceville, New Jersey; f Bloomberg-Kimmel Institute for Cancer Immunotherapy, Sidney Kimmel Comprehensive Cancer Center, Johns Hopkins University, Baltimore, Maryland

**Keywords:** Mesothelioma, PD-L1, Prognostic, Predictive, Biomarker

## Abstract

**Introduction:**

Given the emergence of combination of programmed cell death protein-1 and CTLA4 pathway blockade as effective treatment options in malignant pleural mesothelioma (MPM), there is interest in the extent to which programmed death-ligand 1 (PD-L1) expression may be prognostic of clinical outcomes and predictive of response to anti–programmed death (ligand) 1 (PD-[L]1) therapies.

**Methods:**

MEDLINE and EMBASE electronic databases were searched until November 4, 2020. English-language randomized trials and observational studies that reported clinical outcomes and PD-L1 expression in adult patients (>18 or >20 y) with MPM were included. Forest plots were used to descriptively summarize clinical outcome data across studies.

**Results:**

A total of 29 publications were identified providing data on the research question. Among the studies in which anti–PD-(L)1 therapies were not specified to have been used, 63% (10 of 16) found patients with tumors expressing PD-L1 (typically >1%) to have poorer survival than those with tumors expressing lower levels of PD-L1. Among the studies in which anti–PD-(L)1 therapies were used, 83% (five of six) did not reveal an association between survival and PD-L1 tumor expression. The single study directly comparing outcomes between those treated and untreated with anti–PD-(L)1 therapies across different PD-L1 cutoffs did not identify any differences between the groups.

**Conclusions:**

The quality and consistency of the existing evidence base are currently insufficient to draw conclusions regarding a prognostic or predictive role of PD-L1 in MPM. Furthermore, high-quality studies on this topic are required to support the use of PD-L1 as a biomarker in MPM.

## Introduction

Malignant mesothelioma is an aggressive malignancy arising from mesothelial cells.^1^ It is a relatively rare form of cancer, most often observed in developed countries, with an average incidence rate of 20 per 1,000,000 per year in Europe, 9.9 per 1,000,000 in the United States of America (Surveillance, Epidemiology, and End Results database 1975–2016), 7 per 1,000,000 in Japan, and 40 per 1,000,000 in Australia.^1^^,^^2^ Malignant pleural mesothelioma (MPM) is the most common form of mesothelioma, accounting for approximately 80% of cases and is linked to industrial pollutant and mineral fiber exposure, with approximately 80% of cases linked to asbestos exposure. Although asbestos has been banned in many developed countries for many years, the latency of MPM from exposure to clinical onset is approximately 40 years, and consequently the disease incidence has not yet declined.^1^

In most patients, by the time MPM has been diagnosed, the disease is advanced and there are no surgical options.^3^ The prognosis is poor, with a 5-year survival rate of less than 5% in males and 10% in females.^4^ For many years, the standard of care for systemic treatment of advanced MPM has been combination chemotherapy with cisplatin and pemetrexed, an approach associated with an improvement in median overall survival (OS) of less than 3 months than cisplatin alone.^5^ The approval of nivolumab and ipilimumab combination therapy as a first-line treatment option in adults with unresectable MPM by the U.S. Food and Drug Administration on October 2, 2020, therefore represents a key development in the treatment of MPM, with trial results reporting a statistically significant and clinically meaningful improvement in OS relative to chemotherapy.^6^

There is an urgent and unmet need for noninvasive biomarkers that can better predict patient response to treatments; however, as yet, no predictive biomarkers have been recommended or validated for clinical practice in MPM.^7^ Given the mechanism of action of nivolumab and other checkpoint inhibitors currently under investigation in MPM, the fact that up to 70% of MPM patient specimens tested have been found to be programmed death-ligand 1 (PD-L1) positive^8^ and the role of PD-L1 as a predictive biomarker in other tumor types, there is interest in the role PD-L1 expression assessed by immunohistochemistry (IHC) may play in the relationship between treatment and clinical outcomes in MPM. This article reports the findings of a systematic literature review (SLR) that sought to collate the published literature regarding the relationship between PD-L1 expression, immunotherapy treatment, and clinical outcomes in MPM and to assess the extent to which it can be used to support a role of PD-L1 as a predictive biomarker in MPM.

## Materials and Methods

### Literature Search

An SLR was designed and refined to identify studies relevant to the review objectives. This SLR was conducted using the Preferred Reporting Items for Systematic Reviews and Meta-Analyses guidelines.^9^ An electronic search was performed in EMBASE (OvidSP) and MEDLINE databases using two sets of terms that broadly related to the health condition (MPM) and biomarker (PD-L1) under investigation. Searches were limited to English language, and publication type was limited to interventional studies (including single-arm and randomized trials) and observational studies in humans. No limit was placed on country or time frame. The search strategy was validated by cross-referencing search strategies with previous published SLRs and unpublished focused reviews and by ensuring known studies were identified. A complete copy of the search strategy used for each electronic database is reported in Supplementary Data 1.

### Study Selection and Quality Assessment

After deduplication of the retrieved records, two analysts independently reviewed the results of the literature search. Studies were included or excluded according to the prespecified inclusion and exclusion criteria. Inclusion criteria included adult patients, diagnosed with having MPM of any stage, any line of therapy, randomized trials and observational studies, and clinical outcomes, including OS, progression-free survival (PFS), objective response rate (ORR), and disease control rate (DCR). Pediatric patients (<18 y), nonhuman studies, conference abstracts, case reports, editorials, letters, reviews, and non–English-language studies were excluded. Titles and abstracts were reviewed for all the retrieved records, and full-text articles were obtained for the included records for evaluation in a full-text review against the eligibility criteria. Any disagreements between analysts were resolved through discussion until a consensus was reached. Retrieved studies were critically appraised by a single reviewer for methodological quality using the Cochrane Risk of Bias tool (RoB2)^10^ for randomized controlled trials and the Risk of Bias In Non-Randomized Studies of Intervention (ROBINS) tool^11^ for nonrandomized studies (results reported in Supplementary Data 2). Informed consent was not required for this study, given it included secondary analysis of existing data with no reported patient identifiers or risks to patients.

### Data Extraction and Synthesis

Data from the included studies were extracted by one analyst and internally quality assured by a second independent analyst. Extracted data included study details and methodology; population characteristics (including disease characteristics and PD-L1 expression status); and PD-L1 diagnostic measurement methodology. Key clinical outcomes extracted were OS, PFS, ORR, and DCR. The association between PD-L1 expression status and disease prognosis was investigated. PD-L1 status was dichotomized according to cutoff thresholds reported in the primary studies, and crude and adjusted hazard ratios (aHRs) for key outcomes (OS and PFS) were calculated where data allowed. These results were reported descriptively and presented as forest plots. Where data on outcomes were missing, this was reported as “not reported.” Where reported, confidence intervals (CIs) and *p* values associated with statistical tests for differences in outcomes across PD-L1 groups were extracted. Although these data are used to provide statements regarding the extent to which results of individual studies are “statistically significant,” this is to provide the reader with some idea of the relative precision and magnitudes of the estimated associations and does not consider the impact of other factors that might be considered to influence statistical significance (statistical power, bias, etc.).

## Results

### Literature Search

**Figure 1:**
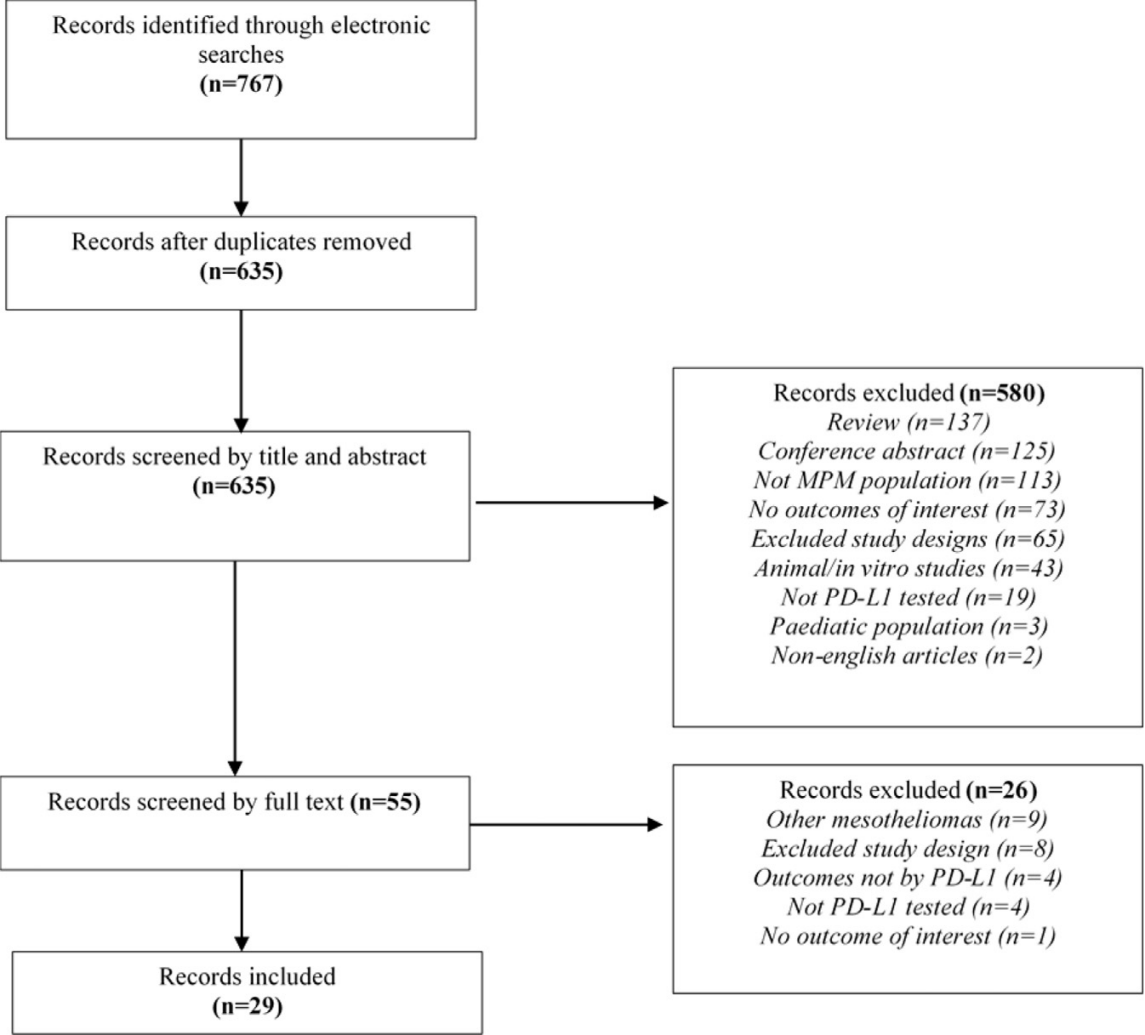
Flowchart of the selection process for studies included in the systematic literature review on the prognostic and predictive effects of PD-L1 in MPM. MPM, malignant pleural mesothelioma; PD-L1, programmed death-ligand 1.

A total of 767 records were identified through electronic database search (full search strategy can be found in Supplementary Data 1). The flow of studies through identification and study selection can be found in Figure 1. After exclusion of 132 duplicate publications, 635 publications were reviewed by title and abstract. Subsequently, 580 publications were excluded, and full-text articles were obtained and screened for 55 publications. A total of 26 publications were excluded for the following reasons: inclusion of peritoneal mesothelioma (*n* = 9), inappropriate study design (*n* = 8), outcomes not reported by PD-L1 expression (*n* = 4), population untested for PD-L1 expression using IHC (*n* = 4), and no outcomes of interest reported (*n* = 1). Finally, 29 publications were included in this SLR.

### Characteristics of Included Studies

A summary of the included studies is presented in Table 1. The studies were published from 2014 to 2020. The sample size of the individual studies ranged from 10 to 329, with this review including 3162 patients overall. The median ages of participants ranged from 49.0 to 77.5 years. Study designs included 24 observational studies (19 retrospective,^5^^,^^8^^,^^12^, ^13^, ^14^, ^15^, ^16^, ^17^, ^18^, ^19^, ^20^, ^21^, ^22^, ^23^, ^24^, ^25^, ^26^, ^27^, ^28^ four prospective,^29^, ^30^, ^31^, ^32^ and one cross-sectional^33^) and five interventional studies (three nonrandomized^34^, ^35^, ^36^ and two randomized study designs^37^^,^^38^). Quality of the included studies was measured using ROBINS-I in the 27 observational and not randomized studies, based on which 12, 13, and two studies were assessed as having low, moderate, and high risk of bias, respectively (see Table 1). The quality of the two randomized studies was assessed using the Cochrane RoB2 tool, both of which were assessed as having some concern of bias.^37^^,^^38^ A total of 23 studies included in this SLR provided data on the association between PD-L1 and OS, six studies reported data on PFS,^12^^,^^18^^,^^22^^,^^29^^,^^31^^,^^36^ four on ORR,^12^^,^^22^^,^^36^^,^^38^ and two on DCR.^22^^,^^38^ Regarding patient populations, eight studies included study populations who were previously treated with first and subsequent lines of chemotherapy.^5^^,^^12^^,^^18^^,^^31^^,^^34^^,^^36^, ^37^, ^38^ There were 11 studies, some reporting on multiple treatments, which included patients treated with immuno-oncology (IO) drugs (*n* = 5 nivolumab [3 mg/kg or fixed dose of 240 mg],^12^^,^^18^^,^^32^^,^^36^^,^^38^ *n* = 5 pembrolizumab [2-10 mg/kg or fixed dose of 200mg],^15^^,^^18^^,^^22^^,^^34^^,^^37^ *n* = 2 ipilimumab [1 mg/kg],^31^^,^^38^ and *n* = 1 durvalumab plus tremelimumab [NR]^35^); four studies, some reporting on multiple treatments, which included patients treated with chemotherapies (*n* = 1 pemetrexed [NR],^29^ *n* = 1 cisplatin plus pemetrexed [NR],^26^ *n* = 2 gemcitabine [1000 mg/m^2^],^26^^,^^37^ *n* = 1 platinum-based chemotherapy [NR],^29^ and *n* = 1 vinorelbine [30, 60, 80 mg/m^2^]^37^); one study which included patients treated with chemotherapy in combination with radiation treatment;^14^ and one chemotherapy in combination with bevacizumab.^29^ The remaining 15 studies did not specify which treatments were received by the included patients; however, given the observational nature of these studies, one can consider the patients in these studies to have received the standard of care available at that time. Given the time frame during which patients were enrolled into these studies (Table 1), the standard of care was unlikely to have comprised IO treatment, and as such, these were categorized as non–IO studies in the remainder of the Results section. A summary of the methods of PD-L1 detection across the included studies can be found in Table 2. PD-L1 was detected using IHC in all included studies; the most frequently used anti–PD-L1 clones were E1L3N, 22C3, 28-8, and SP263.

**Table 1:** Main Characteristics of Included Studies

Study	Country	Enrolment Period	Sample Size	Treatment Received (Dose and Frequency)	Line of Therapy	% Male	Age, y Median (Range)	Clinical Outcomes Reported	Study Quality
**Retrospective observational studies**									
**Cantini et al., 2020**^12^	The Netherlands	NR	107	Nivolumab (3 mg/kg IV, Q2w)	Second	87.0	69.0 (34–84)	OS, PFS, ORR, DCR	Low
**Cedres et al., 2015**^5^	Spain	2000–2014	119	NR	NR	71.4	69.0 (42–90)	OS	Moderate
**Chapel et al., 2019**^13^	USA	NR	125	NR	NR	50.0	69.0 (NR)	OS	Low
**Combaz-Lair et al., 2016**^8^	France	1993–2014	58	NR	NR	77.6	69.0 (58–83)	OS	Low
**De Perrot et al., 2020**^14^	Canada	2008–2016	85	Chemotherapy (NR) + radiation treatment	First	83.0	65.0 (41–82)	OS	High
**Ferdinandus et al., 2020**^15^	NR	2015–2019	27	Pembrolizumab (10 mg/kg IV, Q2w)	≥ Second	85.2	68.0 (51–82)	OS, PFS, ORR	Moderate
**Forest et al., 2018**^16^	France	2002–2017	104	NR	NR	79.0	73.0 (43–92)	OS	Low
**Inaguma et al., 2020**^19^	NR	NR	172	NR	NR	84.3	59.2^a^ (NR)	OS	Moderate
**Inaguma et al., 2018**^17^	USA	NR	175	NR	NR	69.0	59.2^a^ (NR)	OS	Low
**Jiang et al., 2020**^18^	USA	2016–2018	10	Pembrolizumab (200 mg IV, Q3w) or Nivolumab (NR)	≥ Second	66.7	62.3^a^ (NR)	OS, PFS	High
**Kao et al., 2017**^20^	Australia	1992–2007	72	NR	NR	81.0	NR	OS	Moderate
**Mansfield et al., 2014**^21^	USA	1987–2003	106	NR	NR	84.9	NR	OS	Moderate
**Metaxas et al., 2018**^22^	Switzerland, Australia	2015–2017	93	Pembrolizumab (200 mg Q14-21d, 2 mg/kg Q21d, 10 mg/kg Q14d)	First	91.0	68.0 (25–94)	OS, PFS, ORR, DCR	Low
**Muller et al., 2020**^23^	NR	1989–2010	319	NR	First	74.3	64.0 (29–85)	OS	Moderate
**Nguyen et al., 2018**^24^	Australia	2006–2016	58	NR	NR	84.0	73.0 (NR)	OS	Moderate
**Sobhani et al., 2019**^25^	Italy	2005–2016	62	NR	NR	82.0	77.5 (37–92)	OS	Moderate
**Tallón de Lara, et al., 2018**^26^	Switzerland	1999–2009	145	Cisplatin + pemetrexed (Q3w) or gemcitabine (Q3w)	NR	92.0	NR	OS	Moderate
**Thapa et al., 2017**^27^	Australia	1988–2014	329	NR	NR	83.2	NR	OS	Moderate
**Watanabe et al., 2018**^28^	Japan	1992–2016	32	NR	NR	NR	60.5 (34–79)	OS	Low
**Prospective observational studies**									
**Brosseau et al., 2019**^29^	France	2008-2014	214	Pemetrexed (NR) or platinum chemotherapy (NR) with or without bevacizumab (NR)	NR	83.7	66.9 (35–76)	OS, PFS	Moderate
**Cedres et al., 2016**^30^	Spain	2000-2014	27	NR	NR	70.4	68.0 (53–83)	OS	Low
**Disselhorst et al., 2019**^31^	NR	2016-2017	35	Nivolumab (240 mg IV Q2w) + ipilimumab (1 mg/kg IV Q6w up to 4 doses)	≥ Second	86.0	65.0 (37–79)	OS, PFS, ORR, DCR	Low
**Quispel-Janssen et al., 2018**^32^	The Netherlands	2015-2016	34	Nivolumab (3 mg/kg IV biweekly)	≥ Second	82.0	67.0 (50–81)	OS, PFS, ORR, DCR	Low
**Cross -sectional studies**									
**Salaroglio et al., 2019**^33^	Italy	2016-2018	62	NR	NR	73.5	49.0^a^ (52–85)	OS, PFS	Low
**Nonrandomized trials**									
**Alley et al., 2017**^34^	6 countries (NR)	2014-2014	25	Pembrolizumab (10 mg/kg IV, Q2w)	≥ Second	75.0	65.0 (57–73^b^)	OS, PFS, ORR	Moderate
**Chiarucci et al., 2020**^35^	NR	NR	40	Tremelimumab + durvalumab	NR	NR	66.0 (42–83)	OS	Moderate
**Okada et al. 2019**^36^	Japan	2016-2018	34	Nivolumab (240 mg IV, Q2w)	≥ Third	79.0	68.0 (43–78)	OS, PFS, ORR, DCR	Low
**Randomized trials**									
**Popat et al., 2020**^37^	UK, Switzerland, Spain	2017-2018	144	Gemcitabine (1000 mg/m^2^ IV, Q3w) or vinorelbine (30 mg/m^2^, 60 mg/m^2^, or 80 mg/m^2^ IV, Q3w) vs. pembrolizumab (200 mg IV, Q3w)	≥ Second	88.9	70.0 (52–83)	OS, PFS, ORR	Some concern
**Scherpereel et al., 2019**^38^	France	2016	125	Nivolumab (3 mg/kg IV Q2w) with or without ipilimumab (1 mg/kg IV, Q6w)	≥ Second	84.0	NR	OS, PFS, ORR, DCR	Some concern

DCR, disease control rate; NR, not reported; ORR, objective response rate; OS, overall survival; PFS, progression-free survival; USA, United States of America.

a Mean value reported.

b IQR reported.

**Table 2:** PD-L1 Detection Methods From the Included Studies

Study	Detection Method	Primary Antibody					Cutoff Value, %
		Antibody Source	Antibody Type	Antibody Clone	Antibody Dilution	Antibody Company	
**Retrospective studies**							
**Cantini et al., 2020**^12^	IHC	NR	NR	22C3 or SP263	NR	NR	≥1
**Cedrés et al., 2015**^5^	IHC	Rabbit	MAB	E1L3N	1:1200	Cell Signaling Technology, Danvers, MA	≥1
**Combaz-Lair et al., 2016**^8^	IHC	Rabbit	MAB	E1L3N	1:100	Cell Signaling Technology, Danvers, MA	≥1%
		Rabbit	NR	SP142	1:60	Spring Bioscience	≥1
**Chapel et al., 2019**^13^	IHC	NR	NR	22C3	NR	Dako pharmDx, Carpinteria, CA	≥1
				28-8	NR	NR	≥1
**De Perrot et al., 2020**^14^	IHC	NR	NR	28-8	1:200	Abcam Inc., Toronto, Ontario, Canada	≥1%
**Ferdinandus et al., 2020**^15^	IHC	NR	NR	28-8	NR	Abcam	NR
**Forest et al., 2018**^16^	IHC	NR	NR	22C3	1:40	Agilent, Santa Clara, CA	≥1
**Inaguma et al., 2020**^19^	IHC	Mouse	NR	EPR4877	1:200	Abcam	NR
**Inaguma et al., 2018**^17^	IHC	Rabbit	MAB	E13LN	1:200	Cell Signaling Technology, Danvers, MA	≥5
**Jiang et al., 2020**^18^	IHC	NR	NR	NR	NR	NR	≥1
**Kao et al., 2017**^20^	IHC	Rabbit	MAB	E13LN	1:75	Cell Signaling Technology, Danvers, MA	≥5%
**Mansfield et al., 2014**^21^	IHC	Mouse	MAB	5H1-A3	1:300	NR	≥5
**Metaxas et al., 2018**^22^	IHC	Rabbit	MAB	E1L3N	NR	Cell Signaling Technology, Danvers, MA	≥5
**Muller et al., 2020**^23^	IHC	Rabbit	MAB	E1L3N	1:100	Cell Signaling Technology, Danvers, MA	≥1
**Nguyen et al., 2018**^24^	IHC	Rabbit	MAB	SP263	NR	Ventana	≥1
**Sobhani et al., 2019**^25^	IHC	NR	MAB	22C3	1:50	Agilent Dako	NR
**Tallón de Lara et al., 2018**^26^	IHC	NR	NR	E13LN	1:1000	Cell Signaling Technology, Danvers, MA	≥1
**Thapa et al., 2017**^27^	IHC	Rabbit	MAB	E13LN	NR	Cell Signaling Technology, Danvers, MA	≥1
**Watanabe et al., 2018**^28^	IHC	NR	NR	SP142	NR	Ventana Medical Systems, Tucson, AZ	≥1
**Prospective studies**							
**Brosseau et al., 2019**^29^	IHC	NR	NR	E1L3N	1:400	CST or Ozyme	≥1
**Cedres et al. 2016**^30^	IHC	Rabbit	MAB	E1L3N	1:1200	Cell Signaling Technology, Danvers, MA	≥1
**Disselhorst et al., 2019**^31^	IHC	NR	NR	22C3	NR	Agilent pharmDx, Santa Clara, CA	≥1
**Quispel-Janssen et al., 2018**^32^	IHC	NR	MAB	28-8	NR	Agilent Dako, Santa Clara, CA	≥1
**Cross-sectional studies**							
**Salaroglio et al., 2019**^33^	IHC	Mouse	NR	29E.2A3	1:100	BioLegend, San Diego, CA	NR
**Nonrandomized trials**							
**Alley et al., 2017**^34^	IHC	NR	NR	22C3	NR	Dako pharmDx, Carpinteria, CA	≥1
**Okada et al., 2019**^36^	IHC	NR	NR	28-8	NR	Dako	≥1
**Randomized trials**							
**Popat et al., 2020**^37^	IHC	NR	NR	SP263	NR	NR	≥1
				E1L3N	NR	NR	≥1
**Scherpereel et al., 2019**^38^	IHC	NR	MAB	28-8	NR	Dako pharmDx, Carpinteria, CA	≥1
			MAB	SP263	NR	NR	≥1

IHC, immunohistochemistry; MAB, monoclonal antibody; NR, not reported.

### Prognostic Nature of PD-L1 on Clinical Outcomes in MPM

Overall, there were 23 studies providing data on the relationship between PD-L1 expression and OS. Of these, 18 studies reported median OS stratified for individuals classified as having PD-L1–negative (range: 6.1–24.0 mo) and individuals classed as having PD-L1–positive tumors (range: 2.0–27.0 mo). Of the 22 studies that provided a statistical assessment of the comparability of survival distributions, 11 studies did not report significant differences (ranging between *p* = 0.89 to *p* *=* 0.08),^12^^,^^13^^,^^18^^,^^22^^,^^24^^,^^25^^,^^28^^,^^29^^,^^31^^,^^36^^,^^37^ 10 studies reported results consistent with a statistically significantly (ranging between *p* = 0.04 to *p* < 0.0001) higher risk of death in patients with PD-L1–positive tumors compared with PD-L1–negative tumors (hazard ratio [HR] ranging 1.10–3.91),^5^^,^^17^^,^^19^^,^^20^^,^^21^^,^^23^^,^^26^^,^^27^^,^^30^ and one study (include patient treated with IO therapy) reported results consistent with a statistically significant (*p* = 0.007) lower risk of death in PD-L1–positive compared with PD-L1–negative individuals (HR = 0.16 [95% CI: 0.04–0.73]).^31^

Overall, six studies reported median PFS stratified by individuals classified as having PD-L1–negative tumors (range: 1.7–9.5 mo) and individuals classed as having PD-L1–positive tumors (range: 2.5–16.4 mo) 12, 18, 22, 29, 31, 36, 37. Among the five studies that provided a statistical assessment of the difference in PFS, three studies (all including IO-treated patients) reported statistically significant (ranging between *p* = 0.04 and *p* = 0.0018) longer PFS in individuals classed as having PD-L1–positive tumors compared with individuals classed as having PD-L1–negative tumors, whereas three studies (two containing IO-treated patients and one containing non–IO-treated patients) did not identify a statistically significant difference (ranging between *p* = 0.11 and *p* *=* 0.84) between these groups.

### Clinical Outcomes by PD-L1 Expression in Non–IO-Treated Patients

**Figure 2:**
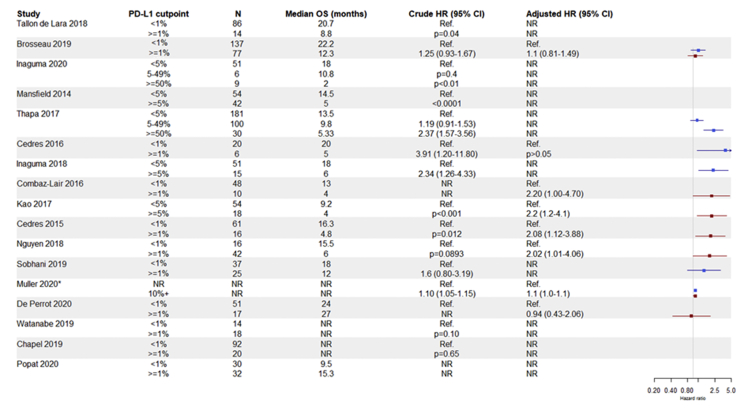
Forest plot displaying crude (blue) and adjusted (red) HRs comparing the hazard of death in individuals classified according to PD-L1 expression. Where HRs are not reported but a *p* value from a relevant statistical test (e.g., log-rank test), these are provided to provide indication of statistical significance of differences in survival distributions. ∗HRs describe increase in hazard for every 10% increase in PD-L1 expression. CI, confidence interval; HR, hazard ratio; NR, not reported; OS, overall survival; PD-L1, programmed death-ligand 1; Ref., reference.

There were 17 studies providing data on the relationship between PD-L1 expression and OS in patients treated with non–IO therapies. Of these, 14 studies reported median OS stratified for individuals classified as having PD-L1–positive (range: 2.0–27.0 mo) and PD-L1–negative tumors (range: 8.0–24.0 mo) (see Fig. 2). Of the 16 studies that provided a statistical assessment of the comparability of survival distributions, six studies did not report significant differences (ranging between *p* *=* 0.87 to *p* = 0.08)^13^^,^^24^^,^^25^^,^^28^^,^^29^ and 10 studies reported results consistent with a statistically significant (ranging between *p* = 0.04 to *p* < 0.0001) higher risk of death in patients with PD-L1–positive tumors compared with those with PD-L1–negative tumors (HR ranging 1.10–3.91).^5^^,^^17^^,^^19^^,^^20^^,^^21^^,^^23^^,^^26^^,^^27^^,^^30^ Studies that reported multivariable models that adjusted for age, sex, disease subtype (e.g. epitheloid, sarcomatoid, and biaphasic subtypes) and other factors consistently identified an association between PD-L1 expression and poor OS. Two studies did not report significant differences (*p* = 0.2 and *p* = 0.55),^14^^,^^29^ whereas six of the studies reported results consistent with a statistically significant (ranging between *p* = 0.04 to *p* = 0.001) higher risk of death in individuals with PD-L1–positive tumors compared with those with PD-L1–negative tumors (HR ranging 1.1–2.2).^5^^,^^8^^,^^20^^,^^23^^,^^24^^,^^30^ Notably, among all the studies with no significant findings, the direction of effect (i.e., median survival or HR) was consistent with poorer survival of patients with PD-L1–positive tumors in all but one of the studies. The effect of different PD-L1 expression cutpoints on OS was explored in six studies.^5^^,^^19^^,^^22^^,^^23^^,^^27^^,^^29^ Three of these studies reported results consistent with a statistically significant increased hazard of death in individuals with greater than or equal to 50% PD-L1 expression compared with those with less than 50% PD-L1 expression (HR = 1.93 [95% CI: 1.27–2.93], *p* = 0.002)^29^ or less than 5% PD-L1 expression (*p ≤* 0.001^27^ and *p* ≤ 0.01^19^).

Two non–IO studies were identified providing data on the association between PD-L1 and PFS in MPM. Brosseau et al.^29^ reported on 448 patients from the Bio-MAPS prospective cohort treated with pemetrexed plus cisplatin and bevacizumab (*n* = 223) or pemetrexed plus cisplatin (*n* = 223) from February 2008 to January 2014. This study reported shorter median PFS in individuals with PD-L1 expression greater than 1% (6.9 mo) compared with individuals with PD-L1 expression less than or equal to 1% (9.5 mo). The aHR (adjusted for disease subtype (epithelioid, sarcomatoid, or biphasic), performance status, smoking, and treatment arm) was consistent with no significant difference (*p* *=* 0.84) in PFS between PD-L1 expression groups (aHR = 0.97 [95% CI: 0.72–1.31]). When a higher PD-L1 cutpoint (50%) was used, a shorter median PFS was reported in individuals with PD-L1 expression greater than 50% (6.2 mo) compared with individuals with PD-L1 expression less than or equal to 50% (9.2 mo). Unadjusted HR supported a significantly lower PFS in the greater than 50% PD-L1 expression groups compared with the less than or equal to 50% PD-L1 group (*p* = 0.001); however, this became not significant when adjusted for disease subtype, performance status, smoking, and treatment arm (*p =* 0.37). The second study reporting PFS data in non–IO-treated patients provided median PFS in chemotherapy-treated patients based on PD-L1 thresholds of 1% and 20% but did not report a statistical assessment of the difference in PFS between these groups.^37^

### Clinical Outcomes by PD-L1 Expression in IO-Treated Patients

**Figure 3:**
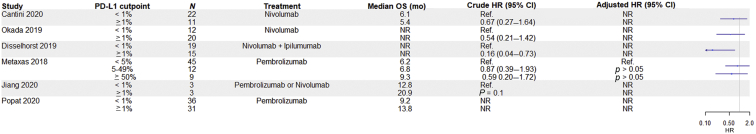
Forest plot displaying crude (blue) and adjusted (red) HRs comparing the hazard of death in individuals treated with anti–PD-1 therapy classified according to PD-L1 expression. Where HRs are NR but a *p* value from a relevant statistical test (e.g., log-rank test), these are provided to provide indication of statistical significance of differences in survival distributions. CI, confidence interval; HR, hazard ratio; NR, not reported; OS, overall survival; PD-1, programmed cell death protein-1; PD-L1, programmed death-ligand 1; Ref., reference.

Six studies reported on the association between PD-L1 expression and OS in patients receiving IO therapies (Fig. 3).^12^^,^^18^^,^^22^^,^^31^^,^^36^^,^^37^ One study^31^ reported results consistent with a statistically significant (*p* = 0.007) lower risk of death in individuals classified with PD-L1–positive compared with PD-L1–negative expressing tumors (HR = 0.16 [95% CI: 0.04–0.73]). Among the other five studies, statistically significant differences in survival were not reported (ranging between *p* = 0.1 and *p* *=* 0.87); however, it is notable that median survival and HR estimates (HR range: 0.54–0.84) consistently suggested a direction of effect consistent with IO-treated patients with PD-L1–positive tumors having longer survival than IO-treated patients with PD-L1–negative tumors.

**Figure 4:**
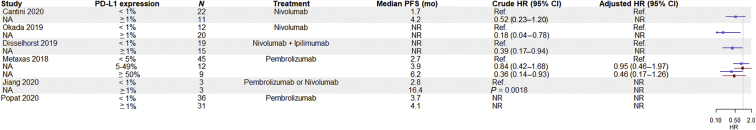
Forest plot displaying crude (blue) and adjusted (red) HRs comparing the hazard of progression in individuals treated with anti–PD-1 therapy classified according to PD-L1 expression. Where HRs are not reported but *p* value from log-rank or Wald test, these are provided to provide an indication of statistical significance of difference in survival distributions. CI, confidence interval; HR, hazard ratio; NA, not applicable; NR, not reported; PD-1, programmed cell death protein-1; PD-L1, programmed death-ligand 1; Ref., reference.

The same six studies also reported data comparing PFS between PD-L1–positive and PD-L1–negative tumor groups treated with anti–programmed cell death protein-1 (PD-1) therapy (Fig. 4).^12^^,^^18^^,^^22^^,^^31^^,^^36^,^37^ Again, all results were directionally consistent with longer PFS in PD-L1–positive tumor groups. Among the five studies that provided a statistical assessment of the difference in PFS, two of the studies reported statistically significant (*p* = 0.0018 and *p* = 0.029) longer PFS in patients with tumors expressing PD-L1 greater than or equal to 1% compared with patients with tumors expressing PD-L1 less than 1%^18^, ^31^, whereas two studies did not identify a statistically significant difference (*p* *=* 0.11 and *p* = 0.449) between these groups.^12^, ^36^ The final study reported statistically significant (*p* *=* 0.04) longer PFS in those with PD-L1 greater than or equal to 50% compared with those with PD-L1 less than 5% (HR = 0.36 [95% CI: 0.14–0.93]); however, this association was no longer observed when an adjusted model was used (HR = 0.46 [95% CI: 0.17–1.26]) to account for baseline differences between groups.^22^

### Clinical Outcomes by PD-L1 Expression and IO Treatment Versus Chemotherapy

One study was identified directly comparing clinical outcomes by IO treatment status in patients with tumors expressing different levels of PD-L1, the randomized controlled PROMISE-Meso trial.^37^ This open-label, 1:1, randomized, phase 3 trial investigated the efficacy of pembrolizumab versus chemotherapy (gemcitabine or vinorelbine) in 144 patients with progressive MPM (after platinum-based chemotherapy). Descriptive results of these studies have been reported in the previous sections; here, we report the results of the formal comparisons made within subgroups. This study reported no difference in OS and PFS between treatment arms in subgroups of patients with PD-L1 less than 1% (OS: HR = 0.96, 95% CI: 0.53–1.75; PFS: HR = 0.76, 95% CI: 0.44–1.30), greater than or equal to 1% (OS: HR = 1.09, 95% CI: 0.57–2.09; PFS: HR = 0.76, 95% CI: 0.44–1.30), less than 20% (OS: HR = 0.96, 95% CI: 0.59–1.55; PFS: HR = 0.95 95% CI: 0.62–1.45), and greater than or equal to 20% (OS: HR = 1.11, 95% CI: 0.36–3.40; PFS: HR = 0.95, 95% CI: 0.62–1.45).

## Discussion

The identification of biomarkers predicting response to immunotherapy with PD-1– and PD-L1–targeted monoclonal antibodies is an area of active investigation and debate across several areas of oncology. This has led to the use of PD-L1 to define treatment-eligible subgroups in several tumor types, including NSCLC, bladder cancer, cervical cancer, and breast cancer. Within MPM, the scientific conversation has been characterized by the reporting of conflicting findings in the literature regarding the relationship between PD-L1 expression, PD-1 or PD-L1–targeted immunotherapy, and clinical outcomes. This SLR seeks to support debate on this topic by providing a comprehensive and updated overview of work in this area.

This SLR identified 29 studies published during 2014 to 2020, reporting on PD-L1 expression and clinical outcomes in patients with MPM. There were 23 that reported data on the association between PD-L1 and OS, whereas six studies provided data on PFS. Among patients receiving non–IO therapy, individuals classified as having PD-L1–positive tumors seemed to have poorer prognosis compared with individuals classified with having PD-L1–negative tumors. In contrast, among patients receiving IO therapy, similar (four studies) or better (one study) survival was observed in individuals classified with having PD-L1–positive tumors compared with individuals classified with having PD-L1–negative tumors. These results are further discussed subsequently.

Across the 14 studies reporting data on OS in which the populations were not specified to have received treatment with PD-1 or PD-L1–targeted therapies, those individuals with tumors expressing PD-L1 seem to have poorer prognosis than those expressing no PD-L1. In contrast, in the six studies that formally compared outcomes by PD-L1 status among individuals known to have been treated with PD-1 or PD-L1–targeted therapies, similar (*n* = 5) or better (*n* = 1) survival in patients with tumors expressing PD-L1 compared with patients without PD-L1–expressing tumors was observed. Taken together, these findings suggest that the baseline prognosis of patients with PD-L1–positive tumors may be poorer than that of patients with PD-L1–negative tumors and that the outcomes observed in the populations treated with PD-1 or PD-L1–targeted therapies may reflect an improvement in the survival of the PD-L1–positive patients to a level similar to or, in some cases, better than that of PD-L1–negative patients.

Although this observation provides some evidence to suggest a prognostic and therapy-dependent predictive role for PD-L1 in MPM, cross-study comparisons may be biased by a variety of factors, and one would ideally draw such conclusions from studies comparing outcomes among patients with similar PD-L1 status who were and were not treated with PD-1 or PD-L1–targeted therapies. Only one study of this nature was identified in this review, in which outcomes in PD-L1–positive and PD-L1–negative patients treated with pembrolizumab were not found to significantly differ from those in chemotherapy-treated patients.^37^ The CheckMate-743 trial, which was published because this review was carried out, provides further data regarding the predictive role of PD-L1 in MPM.^6^ This phase 3, randomized trial of 605 patients with unresectable MPM was the only study after Popat et al.^37^ to prospectively study the role of PD-L1 in MPM. An unpowered exploratory subgroup analysis of the trial focusing on PD-L1 revealed a potentially different OS benefit of nivolumab plus ipilimumab versus chemotherapy in patients with PD-L1 expression greater than or equal to 1% (median OS = 18.0 versus 13.3 mo, HR = 0.69 [95% CI: 0.55–0.87]) versus in those with expression less than 1% (median OS = 17.3 versus 16.5 mo, HR = 0.94 [95% CI: 0.62–1.40]). In the patients randomized to chemotherapy treatment in this trial, consistent with the cross-study observations in this review, survival among patients with tumors expressing PD-L1 greater than or equal to 1% (median = 13.3, 95% CI: 11.6–15.4) seems poorer than that in patients with tumors with PD-L1 less than 1% (median = 16.5, 95% CI: 13.4–20.5). In contrast, survival among patients treated with nivolumab plus ipilimumab with tumors expressing PD-L1 greater than or equal to 1% (median = 18.0) seems similar to that in patients with tumors expressing PD-L1 less than 1% (median = 17.3). Notably, across the studies in this review in which anti–PD-1 therapy was used, the only significant finding of this nature observed was also in a population treated with second or subsequent line of nivolumab plus ipilimumab in patients experiencing disease progression or recurrence after platinum-containing systemic therapy.^31^ Again, the CheckMate-743 findings should be interpreted with caution because this was an unpowered exploratory analysis of unstratified PD-L1 subgroups and requires further study.

The results reported here must be taken in context of the small sizes of the studies included in the review, their observational nature, and the quality of information reported. That is, although the body of data provides some evidence suggestive of a potential prognostic and predictive role of PD-L1 in MPM, it is possible that this results from heterogeneity in population characteristics (such as previous therapies received, lines of therapy received, disease subtype (epithelioid, sarcomatoid, or biphasic), stage, and performance status) and approaches to PD-L1 measurement (such as variations in PD-L1 assays and clones used, PD-L1 positivity cutpoint, cell type assessed [e.g., tumor cells versus immune cells], and time point assessed). No consistent trends in outcomes across these factors were observed. Findings are also contingent on the assumption that the 15 studies in which the treatments received were not specified did not include patients exposed to PD-1 or PD-L1–targeted therapies. This assumption seems plausible based on the nature and time period of the studies, and any exposure to such treatments in these studies that did occur would be expected to be minimal and therefore have a relatively small impact on our conclusions. Variation was also observed in the quality of the included studies, with 12, 13, and 2 studies assessed as having low, moderate, or high risk of bias, respectively.

In addition, variation in PD-L1 cutpoint may be a source of heterogeneity between studies. Studies that have assessed the association between PD-L1 and OS based on higher expression cutoffs reported slightly more consistent results, with 3 of 4 studies^19^^,^^27^^,^^29^ investigating the relationship between PD-L1 greater than or equal to 50% and OS identifying a significantly poorer survival in these patients compared with individuals with PD-L1 less than 5%, whereas smaller not significant differences were observed when comparing those with PD-L1 in the 5% to 49% range to those with PD-L1 less than 5%. Although one of these findings^29^ was no longer significant after adjustment for patient characteristics, these results do suggest that more consistency may be obtained at higher PD-L1 thresholds and that the heterogeneity observed in other studies may be related to the distribution of PD-L1 expression levels observed across the populations. If this is the case, future studies might be better to focus on modeling PD-L1 expression as a continuous rather than a categorical variable. Notably, in the one study that took a more continuous approach to modeling PD-L1 expression,^23^ a significant association (*p* = 0.002) between 10% increments in PD-L1 expression and OS was observed.

A recent 2020 meta-analysis^39^ of the prognostic value of PD-L1 in MPM was conducted, including 16 studies reporting data on the relationship between PD-L1 and OS. This SLR provided updated information, including an additional seven studies regarding the relationship between PD-L1 and OS. Nevertheless, given the limitations of the evidence base, a meta-analysis was not carried out as part of this review and caution must be used when interpreting the results of the 2020 meta-analysis, including many of the studies captured in this review. Although the study reported a pooled HR suggestive of an association between high PD-L1 expression and poorer OS (HR = 1.53 [95% CI: 1.28–1.83]), the finding may be heavily affected by some or all of the heterogeneity described previously.^39^

In conclusion, biomarkers capable of identifying patient groups who will gain most benefit from specific treatments, and therefore guide treatment choice, have the potential to offer significant benefits to both patients and health systems. Nevertheless, it is important that they are only used when the evidence base is strong enough to ensure that their potential benefits do not come at the cost of harming patients. In this regard, although this review has identified evidence to suggest that further investigation of the role of PD-L1 in MPM may be warranted, the current evidence base is not sufficient to influence clinical decision-making at this time. As such, it is suggested that further, high-quality studies focused specifically on the role of PD-L1 in MPM, especially randomized controlled trials comparing clinical outcomes in patients with varying PD-L1 tumor expressions across different treatment arms, are required before any firm conclusions can be drawn regarding its potential role in clinical practice.

## CRediT Authorship Contribution Statement

Bristol-Myers Squibb contributed to the design and reporting of the analysis.

**Aaron Mansfield:** Conceptualization, Writing - original draft, Writing - review & editing.

**Rebecca J. Brown, Cormac Sammon:** Methodology, Investigation, Formal analysis, Data curation, Writing - original draft, Writing - review & editing, Visualization.

**Melinda J. Daumont:** Conceptualization, Methodology, Resources, Writing - review & editing, Supervision, Project administration, Funding acquisition.

**Mike McKenna:** Conceptualization, Methodology, Resources, Writing - review & editing, Supervision, Project administration.

**Jenine K. Sanzari:** Writing - review & editing, Project administration.

**Patrick M. Forde:** Conceptualization, Writing - review & editing.
