# Supplementary material for: The Predictive and Prognostic Nature of Programmed Death-Ligand 1 in Malignant Pleural Mesothelioma: A Systematic Literature Review
Source: JTO Clin Res Rep. 2022 Mar 22;3(5):100315. doi: 10.1016/j.jtocrr.2022.100315 (PMC9062484; doi:10.1016/j.jtocrr.2022.100315)
Supplement: Supplemental Data 1 [file mmc1.docx]

# Supplemental Data 1: Search strategy

MEDLINE

Searched 04/11/20 via OVID interface.
No date, language, or study design limits applied.
Database: Ovid MEDLINE(R) and Epub Ahead of Print, In-Process & Other Non-Indexed Citations and Daily <1946 to November 4, 2020>

Search Strategy:

1 exp Mesothelioma/ (14247)

2 exp Pleural Neoplasms/ (13978)

3 ((malignant or pleural) adj3 mesothelioma$).ti,ab. (10248)

4 MPM.ti,ab. (3202)

5 (pleural adj2 (neoplasm$ or cancer$ or tumour$ or tumor$)).ti,ab. (1581)

6 or/1-5 (25148)

7 B7-H1 Antigen/ or Programmed Cell Death 1 Receptor/ or ("PD-L1" or PDL1 or "PD L1" or "B7-H1" or B7H1 or "B7 H1" or "B7 homolog 1" or "B7 homolog1" or "B7-homolog 1" or "B7-homolog1" or "PD-1" or PD1 or "PD 1").ti,ab. (23648)

8 6 and 7 (135)

**EMBASE**Searched 19/10/20 via OVID interface.
No date, language, or study design limits applied.

Database: Embase <1974 to 2020 Week 42>

Search Strategy:

1 exp mesothelioma/ (23173)

2 exp pleura tumor/ (15353)

3 ((malignant or pleural) adj3 mesothelioma$).ti,ab. (14592)

4 MPM.ti,ab. (5547)

5 (pleural adj2 (neoplasm$ or cancer$ or tumour$ or tumor$)).ti,ab. (2217)

6 or/1-5 (33688)

7 programmed death 1 ligand 1/ or programmed death 1 receptor/ or ("PD-L1" or PDL1 or "PD L1" or "B7-H1" or B7H1 or "B7 H1" or "B7 homolog 1" or "B7 homolog1" or "B7-homolog 1" or "B7-homolog1" or "PD-1" or PD1 or "PD 1").ti,ab. (58613)

8 6 and 7 (618)
618 results
