# Supplementary material for: The Predictive and Prognostic Nature of Programmed Death-Ligand 1 in Malignant Pleural Mesothelioma: A Systematic Literature Review
Source: JTO Clin Res Rep. 2022 Mar 22;3(5):100315. doi: 10.1016/j.jtocrr.2022.100315 (PMC9062484; doi:10.1016/j.jtocrr.2022.100315)
Supplement: Supplemental Data 2 [file mmc2.docx]

# Supplemental Data 2: Risk of Bias Assessment Results

| Author (year) | QA tool used | Risk of bias |
| --- | --- | --- |
| Salaroglio (2019) | ROBINS-I tool | Low |
| Inaguma (2020) | ROBINS-I tool | Low |
| Ferdinandus (2020) | ROBINS-I tool | Moderate |
| Muller (2020) | ROBINS-I tool | Moderate |
| Cantini (2020) | ROBINS-I tool | Low |
| Popat (2020) | Cochrane RoB tool 2 | Some concern |
| Brosseau (2019) | ROBINS-I tool | Moderate |
| Sobhani (2019) | ROBINS-I tool | Moderate |
| Mansfield (2014) | ROBINS-I tool | Moderate |
| Cedres (2016) | ROBINS-I tool | Low |
| Combaz-Lair (2016) | ROBINS-I tool | Low |
| Cedres (2015) | ROBINS-I tool | Moderate |
| Marcq (2017) | ROBINS-I tool | High |
| Forest (2018) | ROBINS-I tool | Low |
| Metaxas (2018) | ROBINS-I tool | Low |
| Quispel-Janssen (2018) | ROBINS-I tool | Low |
| Nguyen (2018) | ROBINS-I tool | Moderate |
| Inaguma (2018) | ROBINS-I tool | Moderate |
| Kao (2017) | ROBINS-I tool | Moderate |
| Alley (2017) | ROBINS-I tool | Low |
| Thapa (2017) | ROBINS-I tool | Moderate |
| Jiang (2020) | ROBINS-I tool | High |
| Chiarucci (2020) | ROBINS-I tool | Moderate |
| Okada (2019) | ROBINS-I tool | Low |
| Tallon De Lara (2018) | ROBINS-I tool | Moderate |
| Watanabe (2018) | ROBINS-I tool | Low |
| Chapel (2019) | ROBINS-I tool | Low |
| Disselhorst (2019) | ROBINS-I tool | Low |
| De Perrot (2020) | ROBINS-I tool | High |
| Scherpereel (2019) | Cochrane RoB tool 2 | Some concern |
